# Management of Two Juvenile Myelomonocytic Leukemia Patients According to Clinical and Genetic Features

**DOI:** 10.4274/tjh.2014.0034

**Published:** 2015-05-08

**Authors:** Özlem Tüfekçi, Hale Ören, Fatma Demir Yenigürbüz, Salih Gözmen, Tuba Hilkay Karapınar, Gülersu İrken

**Affiliations:** 1 Dokuz Eylül University Faculty of Medicine, Department of Pediatric Hematology, İzmir, Turkey

**Keywords:** c-CBL mutation, childhood, Juvenile myelomonocytic leukemia, NRAS mutation

## Abstract

Juvenile myelomonocytic leukemia (JMML) is a rare clonal myeloproliferative disorder of childhood. Major progress has been achieved in diagnosis and the understanding of the pathogenesis of JMML by identifying the genetic pathologies that occur in patients. Mutations of RAS, NF1, PTPN11, and CBL are found in approximately 80% of JMML patients. Distinct clinical features have been reported to be associated with specific gene mutations. The advent of genomic studies and recent identification of novel genetic mutations in JMML are important not only in diagnosis but also in the management and prognosis of the disease. Herein, we present 2 patients with JMML harboring different mutations, NRAS and c-CBL, respectively, with distinct clinical features and different therapeutic approaches.

## Abstract (tr)

Juvenil myelomonositik lösemi (JMML) çocukluk çağında nadir görülen klonal myeloproliferatif bir hastalıktır. Hastalarda genetik patolojiler saptandıkça JMML’nin tanı ve patogenezini anlamada önemli ilerlemeler kaydedilmiştir. Bu hastaların yaklaşık %80’inde RAS, NF1, PTPN11 ve CBL gen mutasyonları bulunmuştur. Belirli klinik bulgular ile spesifik gen mutasyonları arasında ilişki olduğu bildirilmektedir. JMML’de genomik çalışmalardaki gelişmeler ve son yıllarda tanımlanmış yeni genetik mutasyonların saptanması sadece hastalığın tanısı için değil, tedavi ve prognozunda da önem taşımaktadır. Burada NRAS ve c-CBL mutasyonları olan iki JMML’li hasta, belirli klinik bulguları ve farklı tedavi yaklaşımları ile sunulmaktadır.

## INTRODUCTION

Juvenile myelomonocytic leukemia (JMML) is a rare clonal myeloproliferative disorder that accounts for 2%-3% of all pediatric leukemias [1,2,3,4,5]. Clinically, patients generally present with pallor, fever, lymphadenopathy, and splenomegaly. Leukocytosis with monocytosis, circulating myeloid/erythroid precursors, varying degrees of myelodysplasia, and thrombocytopenia are common findings found in peripheral blood [1,2,6,7]. Hypersensitivity of hematopoietic progenitors to granulocyte-macrophage colony-stimulating factor is characteristic of JMML [8]. 

Major progress has been achieved in diagnosis and the understanding of the pathogenesis of JMML by identifying the genetic pathologies that occur in patients. Mutations of RAS, NF1, PTPN11, and CBL, the genes involved in the RAS-MAPK pathway, are found in approximately 80% of these patients [9]. The advent of genomic studies and recent identification of novel genetic mutations in JMML are important not only in diagnosis but also in phenotypic presentation, prognosis, and clinical management of the disease [10,11,12,13,14,15,16]. 

In the past, the only known curative therapy for JMML was hematopoietic stem cell transplantation (HSCT), but recently it was reported that some patients have clinical improvement and long-term survival without any treatment [2,6,7,9,10,12,14,15,16]. Herein, we present 2 patients with JMML harboring different mutations, NRAS and c-CBL, respectively, with distinct clinical features and different clinical courses. Informed consent was obtained.

## CASE PRESENTATION

### Patient 1

A 9-month-old male was admitted with complaints of fever, bloody diarrhea, and recurrent lower respiratory tract infections of 3 months in duration. His physical examination was normal. The complete blood count analysis was as follows: hemoglobin of 9.4 g/dL, white blood cells of 39.7x109/L (with neutrophil predominance), and platelets of 185x109/L. Immunological and serological testing excluded immunodeficiency and viral infections. Bone marrow aspiration showed myeloid lineage predominance with slight dysmyelopoiesis. Cytogenetic analysis of the bone marrow sample revealed a normal karyotype and a negative result for the BCR/ABL fusion gene. During follow-up, he began to suffer from diarrhea, febrile episodes, and painful vasculitic skin lesions with no infectious origin. Mild thrombocytopenia, monocytosis, and myeloid precursors on peripheral blood smear appeared 6 months after his first admission. Analysis of hemoglobin electrophoresis revealed increased hemoglobin F (23%). Diagnosis of JMML was made according to the current WHO diagnostic criteria [17]. The genetic work-up from the peripheral blood sample revealed somatic heterozygous mutation in NRAS exon 2 p61 Q>P and a donor search for HSCT was started immediately after diagnosis. A cytoreductive treatment with 6-mercaptopurine and low-dose cytosine arabinoside was started. Prednisolone at a dose of 2 mg/kg per day was started with possible accompanying autoimmune, autoinflammatory characteristics of the disease. After the 15th day of steroid treatment, his fever, cutaneous lesions, and diarrhea attacks subsided. During follow-up, 6-mercaptopurine, low-dose cytosine arabinoside, and steroids were administered from time to time depending on his clinical features. As soon as a fully matched unrelated donor was found, allogeneic HSCT was performed. He has been in remission for 7 months.

### Patient 2

A 17-month-old female presented with fever, failure to thrive, and recurrent respiratory tract infections of a few months in duration. She was 71 cm tall (<3rd percentile), weighed 7.5 kg (<3rd percentile), and had an occipitofrontal head circumference of 43 cm (<3rd percentile). Physical examination revealed broad forehead, mild hypertelorism, short upturned nose, prominent philtrum, mild retrognathism, pallor, petechiae, hepatomegaly (3 cm below the costal margin), and splenomegaly (8 cm below the costal margin). The complete blood count analysis showed leukocytosis (53.7x109/L), monocytosis (11.1x109/L), anemia (Hb: 8.9 g/dL), and thrombocytopenia (platelets: 46x109/L). Peripheral blood smear showed a leukoerythroblastic picture, dysplastic monocytes, and monocytosis (23%). Bone marrow examination revealed cellular smears with myeloid hyperplasia, features of dysmyelopoiesis, and presence of 3% blasts. Cytogenetic studies of the patient revealed a normal karyotype with absence of the BCR/ABL fusion gene, monosomy 7, or any other chromosomal abnormality. Hemoglobin F level was 3%. JMML was diagnosed according to the WHO criteria [17]. Genetic analysis of the peripheral blood samples revealed heterozygous germline mutation in c-CBL exon 8 p371 Y>H. The same genetic mutation was detected in DNA isolated from the hair follicles, indicating the germline origin of the mutation. Her diagnosis was consistent with the CBL syndrome. The family members were negative for c-CBL mutation. Cytoreductive therapy with 6-mercaptopurine (50 mg/m2 per day, given orally) was started and has continued to date. The hepatosplenomegaly decreased in size and leukocytosis regressed. The disease has been stable for 2 years.

## DISCUSSION AND REVIEW OF THE LITERATURE

JMML is clinically a heterogeneous disease [6]. Although most patients experience an aggressive clinical course and die if not treated with HSCT, there are some patients with better clinical course and spontaneous improvement [1,3,6,7,12,16]. Classically, young age at diagnosis (<2 years), platelet counts above 33x109/L, and hemoglobin F of less than 15% at diagnosis have been identified as favorable prognostic factors [3,18]. In addition to these known prognostic factors, increased molecular knowledge of the molecular pathogenesis of JMML has made it possible to outline clinical characteristics and prognosis of the disease for some mutation types [10,11,12,13,14,15,16]. 

Patient 1 was found to have NRAS mutation. RAS proteins are small GTP-binding signaling molecules that control cell proliferation, survival, and differentiation [19]. The activation of RAS is an essential step in the proliferation of cells for most hematopoietic growth factors [20]. Somatic activating point mutations of NRAS or KRAS genes are found in 20%-30% of patients with JMML [6]. Flotho et al. [21] reviewed the clinical and molecular data of 216 cases collected by the EWOG-MDS group and recommended prompt HSCT for every patient with JMML, except children with Noonan syndrome. There are some studies that reported lower relapse rates and better event-free survival with RAS mutations [22,23]. RAS mutations have been reported to be associated with autoimmune phenomena [11,24,25]. Oliveira et al. [24] reported that NRAS has an immune regulatory function. The clinical syndrome of autoimmune phenomena, lymphocyte accumulation, and somatic mutations in NRAS, previously designated as ALPS type IV, is now reclassified as a new nosologic entity termed RALD, for RAS-associated autoimmune leukoproliferative disease [25,26]. 

Mutations causing alteration in the structure of CBL protein have recently been associated with various myeloid malignancies, including JMML [13,27,28]. Patient 2 was found to have marked growth retardation, some phenotypic features, and germline c-CBL mutation. Mutation in c-CBL exon 8 p371 Y>H is the most commonly encountered mutation detected in CBL syndrome [13,14]. Niemeyer et al. [13] reported that the common p371 Y>H mutation causing alteration in CBL protein induces cytokine-independent growth and constitutive phosphorylation only in hematopoietic cells. There are limited reports in the literature about CBL syndrome, but it is important to note that most of these studies have documented the spontaneous resolution rate of JMML in this group of patients [14,15,16]. On the other hand, these patients with CBL syndrome have also been reported to develop serious vasculopathies later in life as evidenced by optic atrophy, hypertension, cardiomyopathy, or arteritis [14]. As our patient harbored germline c-CBL mutation and had a chance of spontaneous resolution, HSCT was not planned as an initial treatment. 

In conclusion, JMML is phenotypically and genotypically a heterogeneous disease. Despite the aggressive clinical course observed in most patients, some patients may have a mild clinical course. Recent developments in identifying molecular lesions have revealed the importance of genotype-phenotype correlation in this disease, which is critical for tailoring the management. Every patient with a possible diagnosis of JMML should be screened for underlying molecular lesions. 
